# An unexpected role for mixotrophs in the response of peatland carbon cycling to climate warming

**DOI:** 10.1038/srep16931

**Published:** 2015-11-25

**Authors:** Vincent E. J. Jassey, Constant Signarbieux, Stephan Hättenschwiler, Luca Bragazza, Alexandre Buttler, Frédéric Delarue, Bertrand Fournier, Daniel Gilbert, Fatima Laggoun-Défarge, Enrique Lara, Robert T. E. Mills, Edward A. D. Mitchell, Richard J. Payne, Bjorn J. M. Robroek

**Affiliations:** 1 School of Architecture, Civil and Environmental Engineering (ENAC), Ecole Polytechnique Fédérale de Lausanne EPFL, Ecological Systems Laboratory (ECOS), Station 2, 1015 Lausanne, Switzerland; 2 Swiss Federal Institute for Forest, Snow and Landscape Research (WSL), Site Lausanne, Station 2, 1015 Lausanne, Switzerland; 3 Centre d’Ecologie Fonctionelle et Evolutive (CEFE), CNRS – Université de Montpellier – Université Paul-Valéry Montpellier – EPHE, 1919 route de Mende, 34293 Montpellier, France; 4 University of Ferrara, Department of Life Science and Biotechnologies, Corso Ercole I d’Este 32, I-44121 Ferrara, Italy; 5 Université de Franche-Comté – Laboratoire Chrono-Environnement, UMR CNRS/UFC 6249, F-25211 Montbéliard cedex, France; 6 Université d’Orléans, ISTO, UMR 7327, 45071 Orléans, France; 7 BRGM, ISTO, UMR 7327, BP 36009, 45060 Orléans, France; 8 CNRS/INSU, ISTO, UMR 7327, 45071 Orléans, France; 9 University of Neuchâtel, Laboratory of Soil Biology, Rue Emile-Argand 11, CH-2000 Neuchâtel, Switzerland; 10 Jardin Botanique de Neuchâtel, Pertuis-du-Sault 56-58, CH-2000 Neuchâtel, Switzerland; 11 Environment, University of York, Heslington, York, YO10 5DD, UK

## Abstract

Mixotrophic protists are increasingly recognized for their significant contribution to carbon (C) cycling. As phototrophs they contribute to photosynthetic C fixation, whilst as predators of decomposers, they indirectly influence organic matter decomposition. Despite these direct and indirect effects on the C cycle, little is known about the responses of peatland mixotrophs to climate change and the potential consequences for the peatland C cycle. With a combination of field and microcosm experiments, we show that mixotrophs in the *Sphagnum* bryosphere play an important role in modulating peatland C cycle responses to experimental warming. We found that five years of consecutive summer warming with peaks of +2 to +8°C led to a 50% reduction in the biomass of the dominant mixotrophs, the mixotrophic testate amoebae (MTA). The biomass of other microbial groups (including decomposers) did not change, suggesting MTA to be particularly sensitive to temperature. In a microcosm experiment under controlled conditions, we then manipulated the abundance of MTA, and showed that the reported 50% reduction of MTA biomass in the field was linked to a significant reduction of net C uptake (-13%) of the entire *Sphagnum* bryosphere. Our findings suggest that reduced abundance of MTA with climate warming could lead to reduced peatland C fixation.

The vast majority of the Earth’s organisms meet their requirements for carbon (C) and energy either by utilising light to assimilate CO_2_ through photosynthesis (autotrophy), or by the uptake of organic C compounds (heterotrophy). However, some organisms have the potential to combine auto- and heterotrophic C uptake, a strategy termed ‘mixotrophy’^1^. Mixotrophy can be found in vastly different taxa using a wide range of mechanisms^2^. Some vascular plants, for instance, are able to acquire organic carbon by trapping invertebrates in addition to the products of their own photosynthesis^3^,^4^. Several microbial eukaryotes acquire organic carbon by combining predation with inorganic C uptake through the photosynthesis of endosymbiotic algae^5^ or the chloroplasts of photosynthetic prey^6^. Among them, mixotrophic protists are widespread and can exceed 80% of total microbial biomass in some aquatic systems^6^,^7^. Mixotrophic protists can both make an important contribution to primary production^8^,^9^ and play an important role in the decomposition pathway as abundant bacterial and fungal grazers^10^,^11^,^12^. Both of these functions influence the ecosystem C balance, and depending on the relative contribution of phototrophy and heterotrophy, mixotrophs can either increase C uptake or release^13^. A shift towards heterotrophy may reduce primary production and enhance grazing pressure on decomposers whilst a shift towards autotrophy may have the opposite effect.

Mixotrophic protists are increasingly studied in both marine and freshwater ecosystems^14^,^15^ but their contribution to C cycling in semi-aquatic ecosystems, such as peatlands, has been almost entirely overlooked. Peatlands sequester and store large amounts of C (*ca*. 400–600 Gt) in the form of slowly decomposing plant material as peat^16^. Peat-forming mosses (*Sphagnum* spp) provide a habitat for a large diversity of aquatic organisms by maintaining waterlogged conditions. These moss-associated organisms include bacteria, fungi, protists and small-sized metazoa^17^, all of which form a microbial food web that critically determines the cycling of C and nutrients. The tight association between *Sphagnum* and these organisms is referred to as the *bryosphere* (*sensu* Lindo & Gonzalez^18^) (Fig. 1). Mixotrophic testate amoebae (MTA) constitute a large proportion of the microbial food web, often exceeding 70% of the total peatland microbial biomass^19^,^20^. With their contribution to CO_2_ assimilation by the bryosphere and by modifying C cycling of the microbial food web, MTA may be major players in peatland C cycling. However, as yet they are no published data on the contribution of MTA to peatland C cycling and possible impact of climate warming. Even though warming experiments have shown a strong response of testate amoeba communities as a whole to a temperature increase^21^,^22^,^23^,^24^, the responses of the specific group of MTA species have not been evaluated.

Here, we combine a long-term field warming experiment and a laboratory microcosm experiment to determine the effects of warming on the composition of the microbial food web, in particular, MTA, and the potential consequences of such changes for CO_2_ uptake of European peatlands. We hypothesized that warming would have a positive effect on MTA biomass, based on recent findings in freshwater ecosystems^14^. Because of their dual role in C mineralization and C assimilation, increasing MTA biomass could (1) alter the C cycle indirectly through a decrease in microbial decomposer biomass (i.e. higher predation pressure), or (2) increase bryosphere C uptake directly through higher photosynthetic activity.

We investigated the response of MTA, their prey and their competitors (bacteria, fungi, ciliates, heterotrophic testate amoebae, rotifers and nematodes) to five years of warming in a temperate peatland in the Jura Mountains, north-eastern France (46°49′35″N, 6°10′20″E). Warming was simulated using open top chambers (OTCs), which consistently increased annual mean air temperature (*ca.* +0.6 °C) with a maximum during summer (ca. +1.1 °C) and a smaller effect during winter (*ca.* +0.2 °C) (Supplementary Fig. S1). OTCs also led air temperature to occasionally spikes of +2 to +8 °C above controls during the summer (Supplementary Fig. S1). The effect of OTCs on annual mean surface peat temperature (−2 cm) was small (ca. −0.2 °C), but with peaks of +0.2 to +3 °C and sometimes up to +6 °C during the summer (Supplementary Fig. S1), mimicking the predicted effects of global warming in Europe^25^. We did not find any difference in light intensity between the controls (1509 ± 27 μmol of photons.m^−2^.s^−1^) and the OTCs (1496 ± 21 μmol of photons.m^−2^.s^−1^). *Sphagnum* moisture content was reduced in OTCs by about 20%, but only on rare occasions during exceptionally dry periods (see Supplementary Fig. S2). Moisture content in the *Sphagnum* layer (0–5 cm depth) in both control and warmed plots strongly depended on the amount of precipitation rather than temperature (Supplementary Fig. S3). It differed between control and warmed plots only when exceeding a threshold of more than 25 days without precipitation during a period of 2 months (Supplementary Fig. S4). In order to minimize potential moisture effects, we took our samples for microbial community analyses when moisture contents were comparable between OTC and control plots (Supplementary Fig. S5).

In our field experiment, testate amoebae (both heterotrophic and mixotrophic species) were the dominant group of predators comprising 61% of the total predator biomass, while ciliates (<1%), rotifers (27%) and nematodes (10%) were recorded in much lower abundance (Table S1). At the beginning of the field experiment before warming started, over 70% of the testate amoeba biomass was MTA. Warming led to a sharp and significant decline in MTA biomass by −40%, −70% and −55% after one, two and five years of warming, respectively (Fig. 2a; Table 1). The reduced MTA biomass was largely driven by a decline in the three dominant species: *Archerella flavum* (−43% after 5 years of warming), *Heleopera sphagni* (−74%), and *Hyalosphenia papilio* (−50%) while the least abundant species *Amphitrema wrightianum* did not respond (Supplementary Table S1). Our results further showed that the higher the intensity of warming, the more that MTA biomass decreased. The difference of MTA biomass between control and warmed plots (standardized effect size) increased with a higher number of summer days with temperature differences between OTC and control plots exceeding 3 °C (*r* = −0.60, *P* = 0.02; Fig. 3). These results suggest that MTA are particularly sensitive to relatively high temperature increases of comparatively short duration. Hence, the frequency of extreme climatic events might be more important for MTA abundance than an average temperature increase (Supplementary Fig. S6).

Our results contrast with a recent short-term warming experiment showing that mixotrophic chrysophytes (*Ochronomas* sp.) from a freshwater system tended to shift their nutritional mode towards heterotrophy, which was followed by increased growth and abundance^14^. This difference may be related to the different evolutionary origins of mixotrophy in the studied organisms. Unlike mixotrophic chrysophytes studied by Wilken *et al.*^14^, which contain a plastid acquired by ancient secondary endosymbiosis, MTA phototrophy is based on regular endosymbiontic algae acquisition^5^. Algal endosymbionts are susceptible to climate warming and, in particular, heat waves can have important effects on the outcomes of host-symbiont interactions^26^,^27^. For instance, increased temperature can induce the production of reactive oxygen, damaging membranes and proteins of the host. In addition to host damage, it can also lead to the death of the symbiont, further reducing growth and reproduction of host cells, or even causing host cell death^26^. An alternative or additional cause for the reduced MTA abundance reported here could be an impaired symbiont acquisition by MTA under higher temperatures. Heat shocks can limit symbiont transmission success from mother to daughter cells during cell division on mixotrophic ciliates^27^, which use algal-endosymbionts similar to MTAs^5^. Living MTAs have not been observed without their endosymbionts since their first description over 130 years ago^28^. This may suggest that MTA are dependent on their endosymbionts and cannot survive as pure heterotrophs^28^. Therefore, unsuccessful symbiont transmission during cell division under climate warming may be an additional mechanism to explain MTAs decline in warmed plots.

Although we sampled during periods with negligible differences in *Sphagnum* moisture content between the control and the warming treatment, we cannot totally exclude the possibility that the occasionally reduced peat moisture in OTCs may also have contributed to the decline in MTA. Indeed, palaeoecological studies showed that decreases in MTA abundance are usually connected with prolonged period of drought^20^,^29^,^30^,^31^. However, recent findings showed that MTA such as *H. sphagni* (i.e. the most affected MTA species in our study) decreased by 70% on average during the last century, while the water table only slightly fluctuated (13.3–14.7 cm)^32^. These findings were attributed to eutrophication; it can, however, not be ruled out that temperature anomalies recorded during the last century^33^ may have played a role in the aforementioned shifts. This underlines our observations and supports the hypothesis that more frequent temperature extremes during summer may directly reduce MTA abundance in peatlands.

Decreasing MTA abundance may affect bryosphere photosynthesis and/or respiration. As facultative predators, a decline in MTA may lead to cascading effects across the food web^34^,^35^. Although total microbial biomass declined by −12%, −34% and −17% after one, two and five warming years, respectively (Fig. 2b), we did not observe any significant changes in the biomass and/or abundance of bacteria, fungi, ciliates, heterotrophic testate amoebae, rotifers, and nematodes (Fig. 2c–i). Likewise, enzymatic activity as a functional characteristic of the bacterial and fungal communities, and the availability and quality of dissolved organic matter reflecting the resource turnover rate of heterotrophic organisms were not affected by warming (see^36^ for details). Overall, these findings suggest that MTA at our study site appear to acquire most of their carbon by photosynthesis of their symbionts, and not by predation. This is in line with a recent study from a similar peatland using stable isotopes, showing that MTA used different C sources than strictly predator testate amoebae^19^. Collectively, these data indicate that MTA at our study site act as autotrophs rather than heterotrophs, with an important impact on photosynthetic C uptake rather than on the decomposition pathway. However, the relative contribution of MTA to overall bryosphere photosynthesis has never been determined.

In order to quantify the potential impact of MTA decline on photosynthetic C uptake by the bryosphere we designed a microcosm-scale exclusion experiment. Due to the methodological difficulties of distinguishing MTA C fixation from that of *Sphagnum*, we decided to manipulate the abundance of MTA (see methods section for details). We achieved a reduction of MTA biomass similar to that observed in response to warming in the field, allowing an indirect estimation of MTA contribution to bryosphere photosynthesis. Changing MTA abundance without affecting other microbial groups or *Sphagnum* is difficult. Because MTA are known to be unable to survive in the absence of light and because they have short generation times^28^, we modified MTA abundance by manipulating the light regime. Half of the microcosms were placed in the dark for two weeks while the other half received a normal light regime (14 h light/ 10 h dark cycles). Such dark treatment was unlikely to affect *Sphagnum,* as mosses from peatlands are snow-covered for more than half of the year and have been shown to recover full photosynthetic capacity within just a few minutes following re-exposure^37^. We tested *Sphagnum* photosystem II efficiency (F_v_/F_m_) and determined chlorophyll a+b content before and after dark treatment in order to assess whether or not the dark treatment affected *Sphagnum* photosynthesis. We used F_v_/F_m_ here as an indicator for plant health and vigor^38^. Bryosphere maximum PSII efficiency (F_v_/F_m_; ANOVA, *F* = 0.19, *P* = 0.67) and bryosphere chlorophyll a+b content (ANOVA, *F* = 3.78, *P* = 0.10) did not significantly differ between the two treatments (Fig. 4a,b). Chlorophyll content, and to a lesser degree the F_v_/F_m,_ even tended to increase in the dark treatment, probably due to the development of the thylakoid system under dark conditions^39^. This shows that the photosynthetic apparatus of *Sphagnum* was not impaired by the dark treatment and remained perfectly operational.

Before the dark treatment, the abundance and biomass of MTA and microalgae were similar in both treatments (*P* > 0.50, Supplementary Fig. 3) and there was no difference in the bryosphere photosynthetic capacity (A_max, bryo_; *P* > 0.90) or chlorophyll a+b content (*P* > 0.50) (Supplementary Fig. S7). More than 90% of the testate amoebae were mixotrophs, mostly *Archerella flavum* (75% of the testate amoeba biomass) and *Hyalosphenia papilio* (15%). Microalgae were largely dominated by the Zygnematophyceae (i.e. desmids) *Cylindrocystis brebissonii*, while very few cyanobacteria were observed. At the end of the microcosm experiment MTA abundance was 46% lower (ANOVA, *F* = 4.68, *P* = 0.04) and MTA biomass 40% lower (*F* = 5.6, *P* = 0.03) in the dark treatment as compared to the light treatment (Fig. 4d). In contrast, microalgae abundance and biomass did not significantly differ between the treatments (*P* > 0.40; Fig. 4e). Microbial chlorophyll content (MTA plus microalgae) was substantially lower in the dark compared to the light treatment (−71%, *F* = 22.5, *P* < 0.01; Fig. 4f). Photosynthetic capacity of the bryosphere (A_max, bryo_) was significantly lower by 13% after the dark treatment (mean 2.2 mg C g sph^−1^ h^−1^) compared to the light treatment (mean 2.5 mg C g sph^−1^ h^−1^, *F* = 5.52, *P* = 0.03; Fig. 4c). Altogether, these data indicate that the decline in photosynthetic CO_2_ uptake in response to the dark treatment was driven by the decrease in MTA abundance. Photosynthetic capacity of individual microcosms decreased significantly with decreasing MTA abundance (Fig. 5a). In contrast, there was no significant relation between microcosm specific photosynthetic capacity and the abundance of microalgae (Fig. 5b).

From these results we conclude that MTA are likely to make a significant contribution to overall bryosphere C fixation and that a reduction in their biomass may reduce *Sphagnum* photosynthetic capacity. In line with our findings, a recent study estimated that mixotrophic protists contribute about 40% to the total C-fixation in aquatic ecosystems^15^. If the decline in MTA observed in our warming experiment was representative for the consequences of climate warming on peatlands, it would suggest that projected increasing temperatures lead to a significant decrease in peatland photosynthetic capacity, and consequently to lower C-fixation. Such upscaling is presently limited, however, by a lack of detailed and direct physiological measurement of MTA photosynthesis (including field measurements) and its temperature sensitivity^14^. Further investigations should also aim for a more detailed mechanistic understanding of the response of MTA abundance to experimental climate manipulation, in particular to disentangle temperature effects from wetness effects.

Evaluating the responses of microbial communities to climate change and their consequences at an ecosystem scale is extremely challenging because of the vast and largely unexplored diversity of microbiota^40^ and the complexity of their trophic interactions^7^. Our results provide support for a novel hypothesis that mixotrophic testate amoebae (MTA) play an important functional role in the peatland C cycle as primary producers. Peatlands accumulate C when input through photosynthesis exceeds C losses through autotrophic and heterotrophic respiration. Any changes in photosynthetic C assimilation or heterotrophic respiration in response to climate warming may thus modify the capacity of peatlands to sequester and store C^41^. Given that *Sphagnum* mosses cover the ground layer of peatlands and that mixotrophic testate amoebae are closely associated with the moss layer, the potential effects of mixotrophic testate amoebae on C assimilation are probably not negligible at the ecosystem scale. The contribution of mixotrophic testate amoebae to the peatland C cycle has been almost entirely overlooked in the past and has not been specifically considered in previous experiments assessing global change effects on peatlands. This topic will need more attention in the future.

## Methods

### Field experiment: Climate manipulation and sampling

We conducted the experiment at the Forbonnet peatland located in the Jura Mountains, France (46°49′35″N, 6°10′20″E). Mean annual precipitation is ca. 1600 mm and mean monthly temperatures in January and July are −1.4 and 14.6 °C, respectively (meteorological data 2009–2013, Forbonnet Scientific Research Station). A mosaic of lawn and hummock microhabitats characterise the peatland surface. The moss layer in the lawns is dominated by *Sphagnum fallax* (H. Klinggr.), and by *S. magellanicum* (Brid.) and *S. fallax* in the hummocks. Ericoid dwarf shrubs *Calluna vulgaris* (L.)*, Vaccinium oxycoccus* (L.)*, Andromeda polifolia* (Link.), and the graminoids *Eriophorum vaginatum* (L.) and *Carex rostrata* (Stokes.) characterize the vascular plant community.

In April 2008, six hexagonal open top chambers (OTCs; height 50 cm, basal diameter, 250 cm) were installed to passively warm the peatland surface. These warming plots (n=6) are complemented by an equal number of control plots. In each plot, air temperature (10 cm above the *Sphagnum* canopy) was recorded continuously every 30 minutes using thermocouple probes linked to a Campbell^TM^ data-logger. *Sphagnum fallax* samples were collected for microbial analyses at the end of June, coinciding with the annual peak of testate amoeba biomass^42^, in 2008, 2009, 2010 and 2013. *Sphagnum* mosses were collected at ten permanently marked locations in each plot (*ca.* 10 g fresh weight per spot, total weight = ca. 100 g from the upper 3 cm of *Sphagnum* shoots), allowing for repeated sampling over time and avoiding any bias due to spatial heterogeneity^43^. The samples were fixed in 20 mL glutaraldehyde (2%) in the field, and stored at 4°C in the dark. All organisms smaller than 300 μm were extracted and counted following the standard protocol described in Jassey *et al.*^23^. This extract included microbial decomposers (fungi and bacteria), phototrophs such as photosynthetic protists (microalgae) and cyanobacteria, and consumers (ciliates, testate amoebae, rotifers and nematodes). For simplicity, all of the extracted organisms (i.e. prokaryotes, microbial eukaryotes and microfauna) are referred to as the ‘microbial communities’ hereafter.

### Microbial communities analyses

Quantification of the abundance of microalgae, cyanobacteria, ciliates, testate amoebae, rotifers and nematodes, as well as their identification to group (most taxa) and species (testate amoebae) level, was carried out using a 3-mL subsample and inverted microscopy (Utermöhl method; Olympus IX71). For fungi, the number and length of hyphae was quantified. Although it was not possible to identify fungi to a lower taxonomic level, this approach allowed fungal biomass estimation. Flow cytometry (FAC-SCalibur flow cytometer, Becton Dickinson) was used for bacterial counts. A 1-ml sub-sample was diluted with 0.02-μm filtered TE (Tris-EDTA) buffer. Samples were stained with SYBR Green I, II (1/10,000 final conc.) and incubated for 15 min in the dark and run at medium speed (ca 40 μL min^−1^). Fluorescent microbeads (molecular probes) of diameter 1-μm were added to each sample as an internal standard. Epifluorescence microscopy was used to determine the size of bacteria: a 1 mL sub-sample was stained with 50 μL of 4,6 diamino-2-phenylindol (DAPI, 0.2% of final concentration) for 15 min in the dark, filtered through 0.2 μm black membrane filters, and examined at ×1000 magnification. For each sample, 20 photographic grips were observed and the size of bacterial cells measured (ca. 800 cells per sample). The size and biovolume of bacteria was estimated by image analysis. For all other taxa, the biovolume (μm^3^) was calculated based on geometric shapes using dimensions measured under the microscope (length or diameter; width, and height). Total biovolume for bacteria, fungi and each other taxon was then converted to carbon (biomass) using standard conversion factors described in Jassey *et al.*^23^. All biomass data were expressed as micrograms of carbon per gram of *Sphagnum* dry mass (μgC.g^−1^ DM).

### Microcosm experiment: experimental design and sampling

For the microcosm experiment eight paired *Sphagnum fallax* peat cores (n = 16, 15 cm deep and 10 cm diameter) were collected in the Store Mosse National Park (Sweden, 57°17′54 N, 14°00′39 E) in October 2014 and transferred into PVC microcosms. The plant community of this site is very similar to that of the field experiment. *Sphagnum fallax* was the dominant moss while ericoid dwarf shrubs such as *Calluna vulgaris, Vaccinium oxycoccus, Andromeda polifolia*, and the graminoid *Eriophorum vaginatum* characterised the vascular plant vegetation. Likewise, microbial community structure at both sites were similar (Supplementary Fig. S8), and most importantly, the communities of microalgae and testate amoebae were dominated by the same species.

In the laboratory, each pair of microcosms was divided and assigned to a light or dark treatment (eight replicates per treatment). All cores received 100 mL of standardized nutrient solution, and throughout the experiment the water level was maintained constant (6 cm below the top of the moss carpet). For both treatments the cores were acclimated for 15 days at 23 °C day and 20 °C night temperature, and a light cycle of 14 h/10 h (light/dark). The light level was set to a constant 600 μmol of photons m^−2^ s^−1^ reflecting the optimum light intensity for *Sphagnum* photosynthesis, which was determined through light response curves at the beginning of the experiment (Signarbieux, unpublished result). After 15 days of acclimation, we randomly sampled 10 g of fresh *Sphagnum* capitula (top 3 cm, avoiding core edges) in each core and fixed these in 20 mL of glutaraldehyde (2% final concentration) for mixotrophic testate amoebae and non-MTA phototrophic protists analyses (method described above). Bryosphere (*Sphagnum* + microalgae + MTA) photosynthetic capacity (A_max,bryo_) and moss chlorophyll content were quantified. After the initial acclimation period half of the samples were placed in the dark (maintaining the same temperature and water table as light conditions) and the other half continued to receive light at the same levels. After a further two weeks samples were again collected and the following measurements were taken: abundance and biomass of MTA and photosynthetic protists, A_max,bryo_, bryosphere maximum PSII efficiency (F_v_/F_m_), and chlorophyll content (plant and microbial content; see method below).

### Bryosphere ecophysiology

We measured bryosphere photosynthesis on two to three (depending of the size) entirely green *Sphagnum* capitula (top 3 cm) at optimal water content (>90% by harvesting 30 minutes after rewetting). A_max, bryo_ was measured with an open path infrared gas analyser (IRGA) system connected to a 2.5 cm^2^ PLC-6 chamber (CIRAS-2, PP-Systems, Amesbury, USA) under optimum conditions for light (600 μmol of photons m^−2^ s^−1^), 20 ± 1 °C, CO_2_ concentration of 380 ± 2 ppm, and relative air humidity ranged between 60 and 70%. Chlorophyll *a* fluorescence was recorded with a portable pulse amplitude fluorometer (PAM-2500, Heinz Walz GmbH, Effel trich, Germany). F_v_/F_m_ was recorded and calculated according to Maxwell and Johnson^44^ once the capitula were dark-adapted for 30 min using a 2030B leaf-clip holder (Heinz Walz GmbH, Effel trich, Germany). Immediately after measurements *Sphagnum* capitula fresh weight (FW) was determined, and then they were freeze-dried to constant weight (DW). A_max, bryo_ was expressed per unit dry weight as mg of CO_2_ per gram of DW per hour (mgC g^−1^ h^−1^).

Bryosphere chlorophyll content was determined from a 20 mg subsample of homogenized freeze-dried capitula (top 3 cm). For microbial chlorophyll content, living photosynthetic cells (mainly MTA and microalgae) were extracted from the remaining *S. fallax* capitula at the end of the experiment by successively rinsing *Sphagnum* (top 3 cm) six times in 200 mL of distilled water, with subsequent filtration at 150 *μ*m (nylon filters, Millipore) and 5.0 *μ*m pore size (membrane filter Whatman). For *Sphagnum* chlorophyll, freeze-dried capitula subsamples were incubated on a platform shaker (100 rpm) in 8 ml 96% ethanol and membrane filters with microbial cells in 8 mL 90% acetone for 15 hrs. Acetone was preferred for microorganisms because it gives very sharp chlorophyll absorption peaks^45^. Samples were vortexed and centrifuged (2500 rmp, 5 min) and 300 μl of the supernatant pipetted into a quartz 96-well plate in triplicate. Absorbance of the extract and triplicated blanks (96% ethanol and 90% acetone as discussed above) was measured at 470.0, 648.6, 664.2 and 750 nm for capitula and 630, 647, 663 and 750 nm for microorganisms on a SynergyMx Microplate Reader (BioTek). The latter absorbance reading (750 nm) only served to correct for impurities. Chlorophyll *a* and *b* content of capitula was then calculated following Lichtenthaler^46^ and expressed as mg of chlorophyll g^−1^ capitula dw. Microbial chlorophyll *a* and *b* content was calculated following Humphrey and Jeffrey^47^ and expressed as μg of chlorophyll per gram of *Sphagnum* dry weight (μgchl. g^−1^ dw). Only absorbance values with <5% deviance between triplicates were accepted for these calculations. In case a sample did not meet this criterion, the extraction procedure was repeated. Contact with light was avoided throughout the whole procedure.

### Numerical analyses

All data were tested for normality and transformed if necessary. We used linear mixed effects models to test the effects of warming and date of sampling (fixed effects) on overall microbial biomass, and the biomass of bacteria, fungi, photosynthetic protists, cyanobacteria, ciliates, testate amoebae, rotifers and nematodes while accounting for the temporal repeated measurements in each plot on the four dates. All models were fitted including plot nested with date as a random effect on the intercept to correct for the inflation of the residual degrees of freedom that would have occurred if we were using repeated measurements as true replicates^48^. The *nlme* package in R was used to run these models^48^. We also used linear mixed effects models to test the effect of light (fixed effects) on the abundance and biomass of MTA and photosynthetic protists, microbial and *Sphagnum* chlorophyll content, A_max, bryo_, F_v_/F_m_, while accounting for the block effect (paired peat cores). We used the standardized effect size index (SES) to estimate the response of MTA to global warming each year. SES was calculated as: observed response of MTA in warmed plots – observed response of MTA in control plot/standard deviation observed response of MTA in control plot. SES was then analysed as a function of the number of summer days with temperature differences between control and warmed plots higher than 3 °C using linear mixed effect models with temperature differences as fixed effect and plot nested with date as a random factor.

## Additional Information

**How to cite this article**: Jassey, V. E. J. *et al.* An unexpected role for mixotrophs in the response of peatland carbon cycling to climate warming. *Sci. Rep.* **5**, 16931; doi: 10.1038/srep16931 (2015).

## Supplementary Material

Supplementary Information

## Figures and Tables

**Figure 1:**
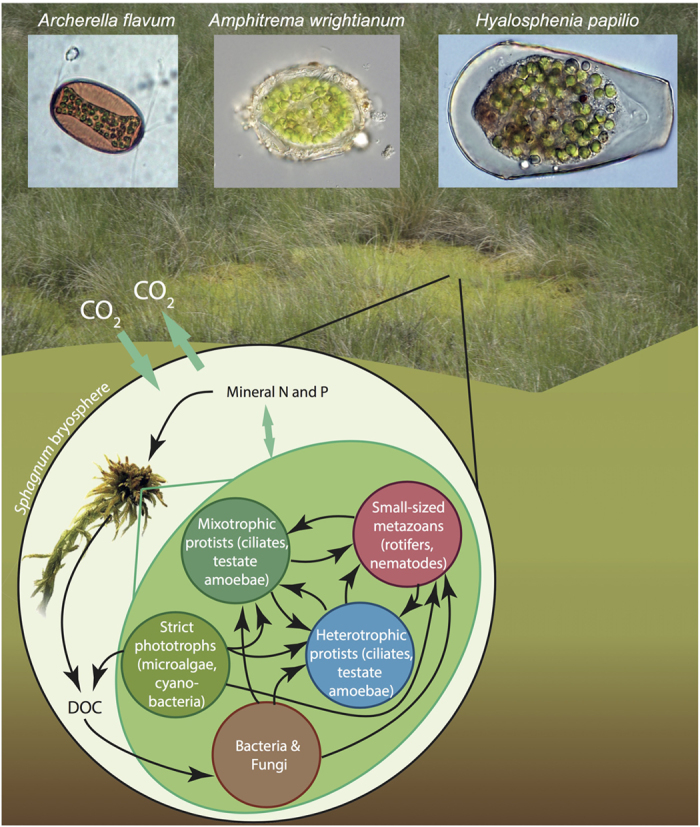
Bryophyte-microbial food web system in peatlands. CO_2_ fixation within the bryosphere is performed by *Sphagnum* moss, photosynthetic protists and mixotrophic protists, as well as cyanobacteria. Mixotrophic protists and heterotrophic protists are involved in numerous trophic interactions influencing the decomposition of dissolved organic carbon (DOC) by bacteria and fungi, and the transfer of energy and nutrients among the various components of the microbial food web. These interactions contribute to the control of the bryosphere C balance. The representation is strongly simplified as it does not show all of the potential trophic relations with microfauna and ignoring a number of other roles of protist communities. Adapted from^7^,^17^,^49^.

**Figure 2:**
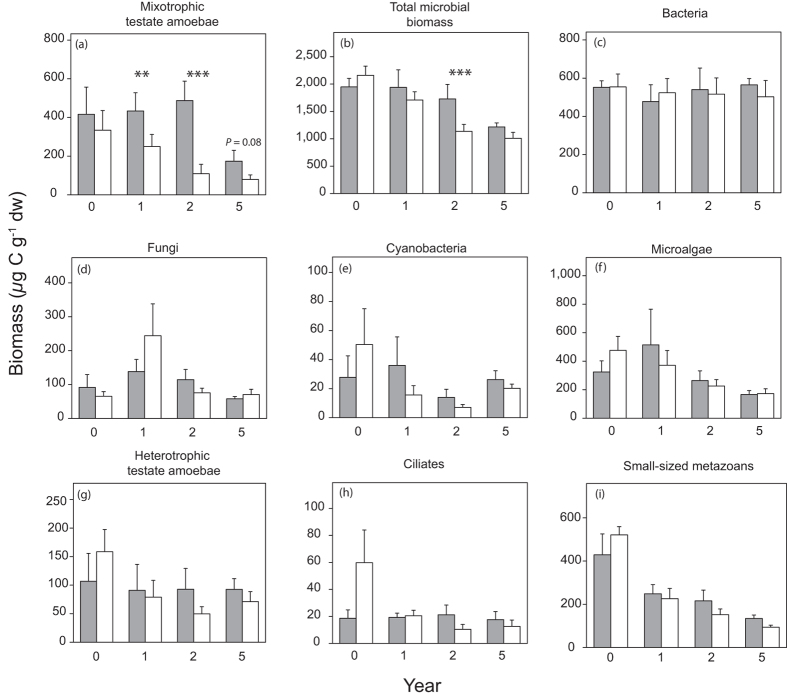
Biomass of different microbial groups in the food web associated to *Sphagnum* mosses from control and warmed plots over five years of experimental field warming (Forbonnet peatland, France). The effect of temperature increase was tested on mixotrophic testate amoeba biomass (mean ± SE) (**a**) total microbial biomass (**b**) bacteria (**c**) fungi (**d**) cyanobacteria (**e**) microalgae (**f**) heterotrophic testate amoebae (**g**) ciliates (**h**) and small-sized metazoans (rotifers and nematodes) (**i**). Grey bars indicate ambient treatment (control) and white bars warmed treatment. Asterisks indicate significant differences between control and warmed plots for each year separately. *P < 0.05; **P < 0.01; ***P < 0.001.

**Figure 3:**
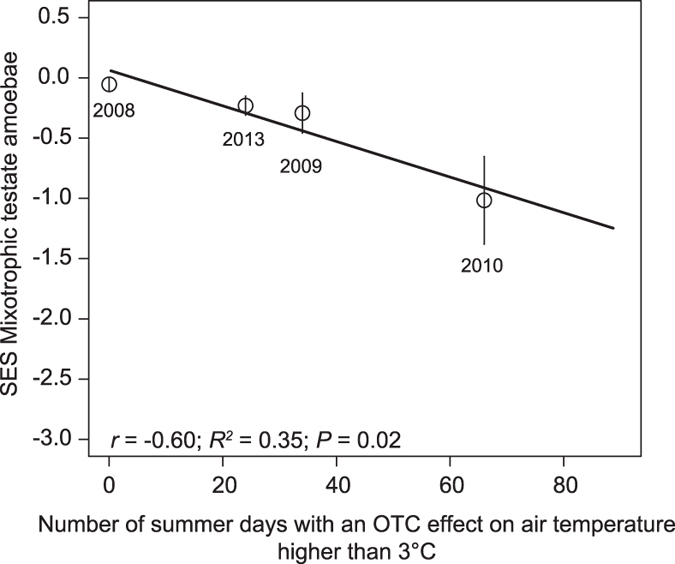
Relationship between the response of mixotrophic testate amoebae (MTA) to warming and the magnitude of OTC warming. Relationship between the MTA standardized effect size (MTA biomass in OTCs –MTA biomass in controls/standard deviation in controls) as a function of the number of summer days with OTC effects higher than 3 °C (mean OTC temperature minus mean control temperature). n = 24; *R*^*2*^ = 0.35; *r* = −0.60; *P* = 0.02; *r* is the coefficient of correlation from linear mixed effect model.

**Figure 4:**
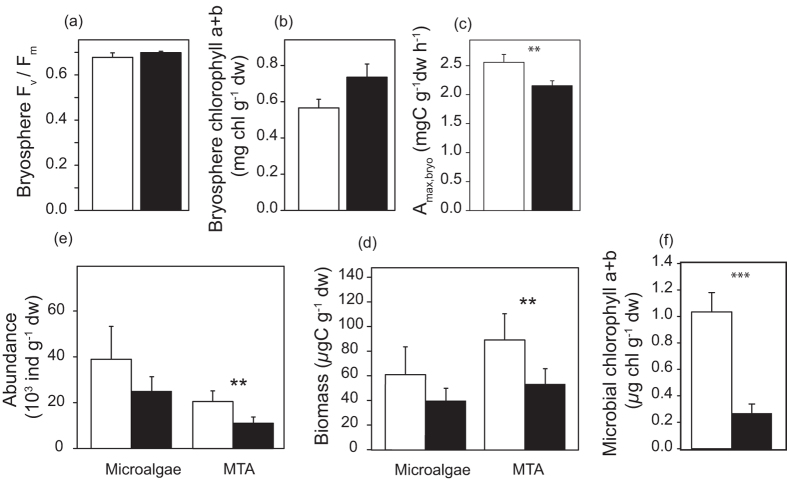
Response of the bryosphere components (*Sphagnum* and associated mixotrophic testate amoebae and microalgae) to full light (PPFD of 600 μmol m^−2^ s^−1^) and dark treatments (no light) in microcosms (mean ± SE). The effect of light conditions was tested on bryosphere photosynthetic capacity (A_max, bryo_) (**a**) bryopshere maximum efficiency of PSII (F_v_/F_m_) (**b**) and bryosphere chlorophyll *a*+*b* content (**c**) microalgae (left) and mixotrophic testate amoeba (right) abundance (**d**) microalgae (left) and mixotrophic testate amoeba (right) biomass (**e**) microbial chlorophyll *a*+*b* content (**f**). White bars indicate light treatment and black bars dark treatment. Asterisks indicate significant differences between light and dark treatment. *P < 0.05; **P < 0.01; ***P < 0.001.

**Figure 5:**
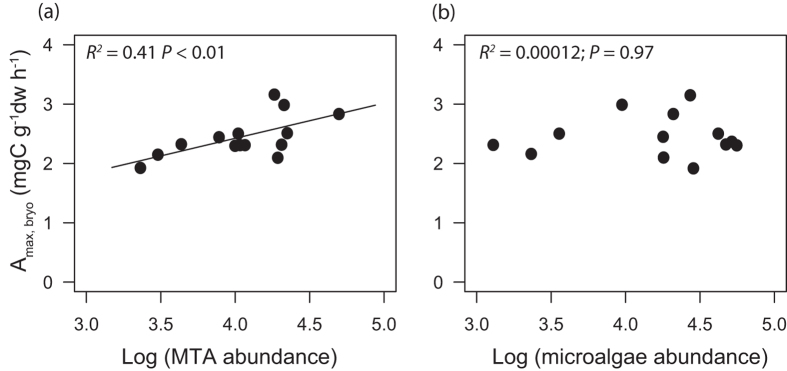
Bryosphere photosynthetic capacity (A_max, bryo_). A_max, bryo_ is shown either as a function of the abundance of mixotrophic testate amoebae (log-transformed) (**a**) or as a function of microalgae (log-transformed) (**b**).

**Table 1:** ANOVA table of *F* and *P* values on the effect of sampling date (D), warming treatment (W), and possible interactions on functional groups of organisms of a peatland microbial food web (field experiment).

	Date (D)		Warming (W)		W × D	
	*F*	*P*	*F*	*P*	*F*	*P*
Total biomass	29.32	**<0.001**	8.01	0.02	0.72	0.4
Microalgae	**6.61**	**0.01**	0.05	0.94	0.06	0.81
Cyanobacteria	0.96	0.33	0.08	0.78	0.37	0.55
Ciliates	3.93	0.055	0.70	0.42	3.41	0.07
Heterotrophic testate amoebae	2.30	0.14	0.03	0.86	1.30	0.26
Mixotrophic testate amoebae	**21.1**	**<0.001**	**8.04**	**0.02**	0.06	0.79
Small-sized metazoans (rotifers and nematodes)	**34.94**	**<0.001**	0.14	0.72	0.47	0.49
Bacteria	1.94	0.17	0.13	0.72	0.05	0.94
Fungi	0.001	0.97	0.03	0.87	0.41	0.52

Bold characters indicate significant effects (*P*-value < 0.05).
