# Supplementary material for: An unexpected role for mixotrophs in the response of peatland carbon cycling to climate warming
Source: Sci Rep. 2015 Nov 25;5:16931. doi: 10.1038/srep16931 (PMC4658499; doi:10.1038/srep16931)
Supplement: Supplementary Information [file srep16931-s1.pdf]

**An unexpected role for mixotrophs in the response of peatland carbon cycling to climate warming**

Vincent EJ Jassey, Constant Signarbieux, Stephan Hättenschwiler, Luca Bragazza, Alexandre Buttler, Frédéric Delarue, Bertrand Fournier, Daniel Gilbert, Fatima Laggoun-Défarge, Enrique Lara, Robert TE Mills, Edward AD Mitchell, Richard J Payne, Bjorn JM Robroek

**Supporting information**

1 supplementary tables, 8 supplementary figures and additional references

**Supplementary Table S1**| Biomass (mean ± SD) and feeding habit of predominant consumers in the microbial food web. B = bacteria; F = fungi; A = algae and cyanobacteria; C = ciliates; R = rotifers; TA = testate amoebae; N = nematodes *;* Feeding habit based on personal observations under microscope and literature [1-9].

| | | **2008** | | | | **2009** | | | | **2010** | | | | **2013** | | | | **Feeding habit** |
|---|---|---|---|---|---|---|---|---|---|---|---|---|---|---|---|---|---|---|
| | | **AMB** | | **WAR** | | **AMB** | | **WAR** | | **AMB** | | **WAR** | | **AMB** | | **WAR** | | |
| | | mean | SD | mean | SD | mean | SD | mean | SD | mean | SD | mean | SD | mean | SD | mean | SD | |
| Nematodes | Unidentified species | 68.2 | *18.6* | 74.9 | *8.1* | 89.6 | *26.1* | 70.6 | *27.3* | 82.9 | *36.8* | 54.6 | *13.6* | 24.6 | *6.1* | 21.9 | *4.7* | B, A, F, TA |
| Ciliates | *Uronema sp.* | 0.3 | *0.1* | 0.2 | *0.1* | 0.1 | *0.1* | 0.1 | *0.1* | 0.1 | *0.3* | 0.0 | *0.0* | 0.1 | *0.1* | 0.1 | *0.0* | B |
| | *Platyophrya sphagni* | 1.5 | *1.7* | 2.2 | *2.9* | 2.3 | *1.1* | 2.4 | *0.7* | 2.7 | *2.7* | 2.2 | *2.5* | 1.1 | *0.7* | 1.7 | *2.0* | B, A |
| | *Paramecium bursaria* | 1.1 | *0.7* | 15.2 | *23.5* | 2.1 | *2.9* | 1.5 | *2.4* | 16.5 | *15.7* | 6.5 | *6.9* | 8.0 | *5.1* | 6.7 | *5.1* | B, A |
| Rotifers | Bdelloidea | 253.8 | *171.1* | 309.9 | *36.9* | 67.7 | *29.5* | 55.4 | *34.8* | 65.3 | *77.0* | 30.7 | *12.5* | 33.9 | *10.3* | 29.1 | *5.2* | B, A |
| | *Colurella colurela* | 12.4 | *30.4* | 0.0 | *0.0* | 80.4 | *59.5* | 58.3 | *40.3* | 27.1 | *70.5* | 9.1 | *8.9* | 7.1 | *8.0* | 4.4 | *5.2* | B, A, C |
| | *Habrotrochoa angusticollis* | 43.3 | *29.3* | 59.9 | *25.4* | 12.3 | *3.8* | 18.5 | *19.8* | 12.8 | *22.1* | 14.8 | *8.2* | 42.8 | *36.7* | 7.0 | *6.8* | B, A |
| | *Lepedella punctata* | 0.7 | *0.4* | 0.6 | *0.3* | 0.5 | *0.4* | 0.3 | *0.1* | 0.2 | *0.1* | 0.2 | *0.1* | 1.9 | *1.4* | 1.0 | *1.1* | B, A |
| | *Lecane quadridentata* | 18.2 | *16.7* | 13.1 | *6.7* | 4.0 | *9.2* | 12.6 | *10.2* | 3.0 | *3.8* | 1.7 | *1.6* | 2.2 | *2.7* | 0.2 | *0.3* | B, A |
| Testate amoebae | *Archerella flavum (mixotroph)* | 7.1 | *3.2* | 6.6 | *3.3* | 15.1 | *1.8* | 10.1 | *5.5* | 11.4 | *8.8* | 5.3 | *5.3* | 4.7 | *4.0* | 2.7 | *3.0* | B |
| | *Amphitrema wrightianium (mixotroph)* | 8.7 | *8.0* | 6.2 | *5.1* | 0.9 | *2.1* | 9.5 | *15.4* | 1.3 | *1.9* | 0.9 | *1.6* | 1.6 | *2.8* | 0.8 | *1.2* | B |
| | *Assulina muscorum* | 2.7 | *2.9* | 2.9 | *2.9* | 4.1 | *2.4* | 5.2 | *4.5* | 4.2 | *3.7* | 4.4 | *7.6* | 2.0 | *1.8* | 2.9 | *2.0* | B |
| | *Assulina seminulum* | 0.1 | *0.2* | 4.7 | *5.1* | 4.2 | *0.2* | 5.0 | *3.5* | 11.6 | *13.5* | 4.5 | *4.2* | 2.9 | *2.2* | 3.8 | *3.0* | B, A, F |
| | *Euglypha compressa* | 0.0 | *0.0* | 0.0 | *0.0* | 0.9 | *1.4* | 0.3 | *0.7* | 3.9 | *5.6* | 0.1 | *0.2* | 1.6 | *1.7* | 1.3 | *2.5* | B, A, F |
| | *Euglypha strigosa* | 4.0 | *3.2* | 2.4 | *2.8* | 0.7 | *40.5* | 1.6 | *3.1* | 1.3 | *1.5* | 0.1 | *0.3* | 1.5 | *1.6* | 2.4 | *2.4* | B, A, F |
| | *Heleopera sphagni (mixotroph)* | 78.3 | *90.1* | 88.0 | *106.9* | 58.1 | *16.9* | 38.9 | *27.4* | 155.8 | *163.0* | 13.1 | *19.1* | 38.0 | *32.5* | 10.1 | *10.4* | A, F, C, TA, R, N |
| | *Hyalosphenia elegans* | 11.6 | *19.7* | 25.8 | *19.7* | 15.2 | *212.6* | 9.2 | *6.7* | 9.6 | *17.8* | 4.1 | *6.4* | 1.3 | *0.8* | 0.3 | *0.6* | A, F |
| | *Hyalosphenia papilio (mixotroph)* | 322.1 | *286.3* | 233.2 | *176.8* | 359.3 | *2.6* | 191.4 | *123.6* | 318.6 | *254.3* | 89.6 | *108.3* | 130.9 | *109.0* | 66.3 | *50.8* | A, F, C, TA, R, N |
| | *Nebela collaris* | 0.0 | *0.0* | 0.0 | *0.0* | 1.6 | *1.5* | 0.0 | *0.0* | 1.5 | *3.6* | 0.0 | *0.0* | 8.6 | *12.3* | 6.2 | *7.3* | A, F, C, R |
| | *Physochila griseola* | 3.1 | *5.0* | 13.2 | *13.0* | 1.2 | *0.2* | 4.0 | *3.8* | 5.6 | *5.7* | 1.6 | *2.1* | 0.7 | *1.1* | 0.0 | *0.0* | A, F, C |
| | *Nebela pernardiana* | 0.1 | *0.1* | 0.2 | *0.3* | 0.1 | *19.3* | 0.0 | *0.0* | 0.0 | *0.0* | 0.0 | *0.0* | 0.0 | *0.0* | 0.0 | *0.0* | A, F, C, TA, R, N |
| | *Nebela tincta* | 57.7 | *78.9* | 80.1 | *86.1* | 12.3 | *29.9* | 15.0 | *22.2* | 13.5 | *22.1* | 14.5 | *24.8* | 12.1 | *15.6* | 16.5 | *22.0* | A, F, C, R |
| | *Nebela tincta var. major* | 0.0 | *0.0* | 0.0 | *0.0* | 14.5 | *0.5* | 16.7 | *31.7* | 17.9 | *30.1* | 6.2 | *11.3* | 17.8 | *20.8* | 12.9 | *9.4* | A, F, C, R, N |

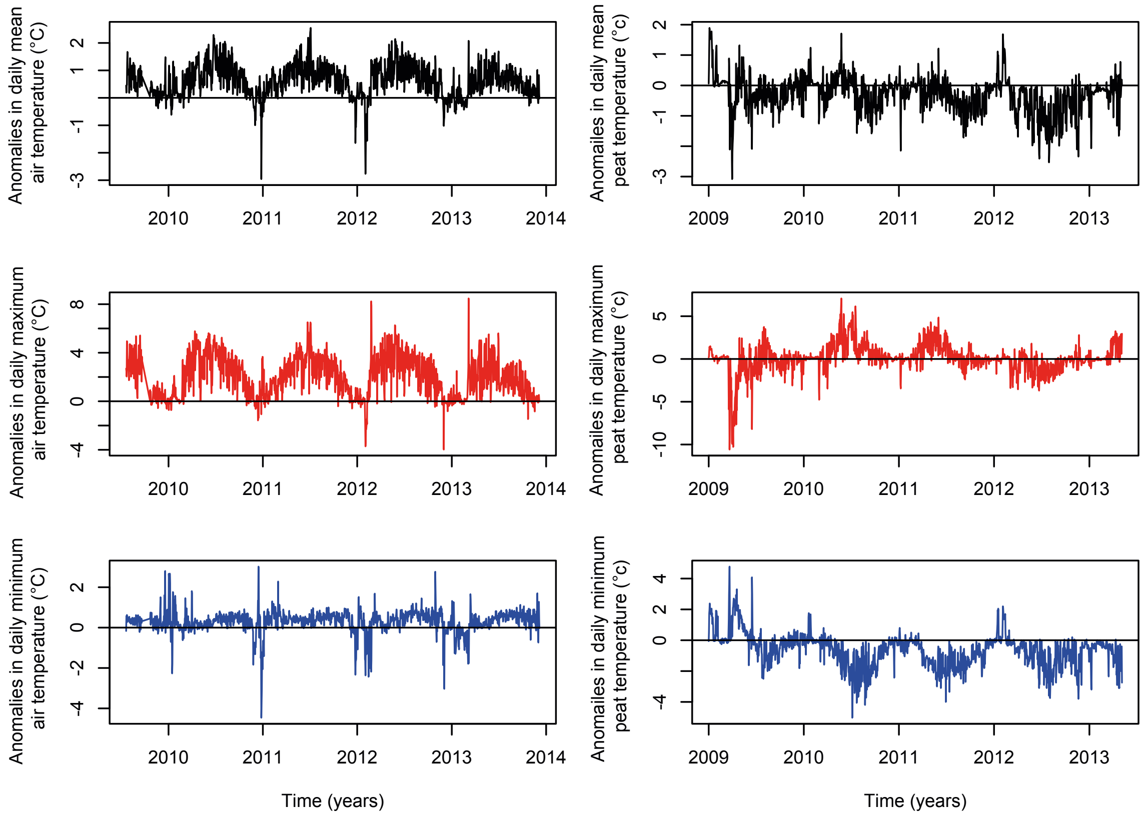


**Supplementary Figure S1| Differences between temperatures measured in OTC and control plots in the field.** On the left, differences in daily mean (top panel), maximum (intermediate panel) and minimum (bottom panel) air temperatures (measured 10 cm above *Sphagnum* carpet) are shown. On the right, differences in daily mean (top panel), maximum (intermediate panel) and minimum (bottom panel) peat temperatures (measured at -2 cm below the *Sphagnum* carpet) are shown.

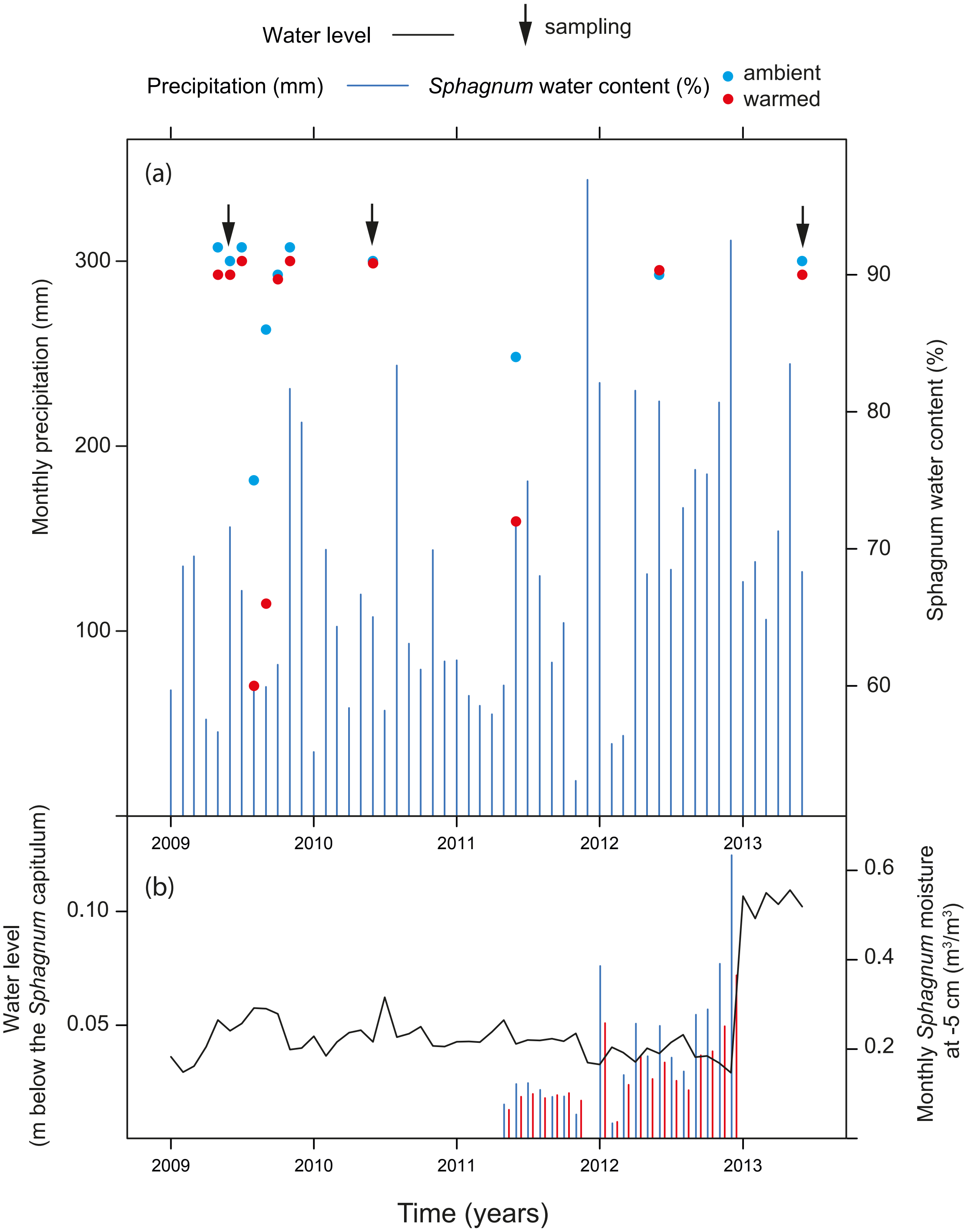


**Supplementary Figure S2| Summary of wetness conditions in the field experiment.** (a) Monthly precipitation (mm) and *Sphagnum* water content (%) in control and warmed plots. (b) Water level (m; mean of two piezometers) and *Sphagnum* moisture at -5 cm depth ($m^3/m^3$) in control and warmed plots.

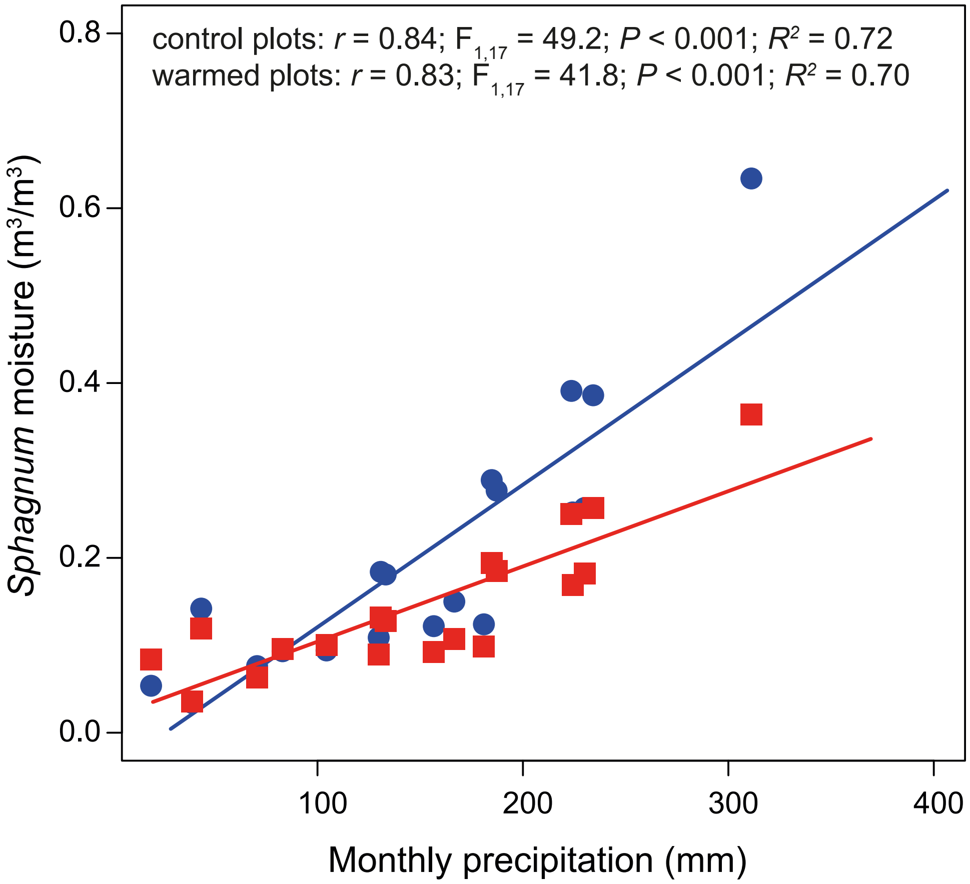


**Supplementary Figure S3| Relationships between *Sphagnum* moisture (-5 cm depth) cm and monthly precipitation in 2011 and 2012.** *r* is the Pearson's correlation coefficient. This figure shows that precipitation are the main driver of *Sphagnum* moisture in both control and warmed conditions. Overall, precipitation explain 63% of *Sphagnum* moisture variations ($F_{1,35} = 97.6$, $P < 0.001$) and temperature 14% ($F_{1,35} = 21.7$, $P < 0.001$) (ANOVA). Blue circles are control plots and red squares warmed plots.

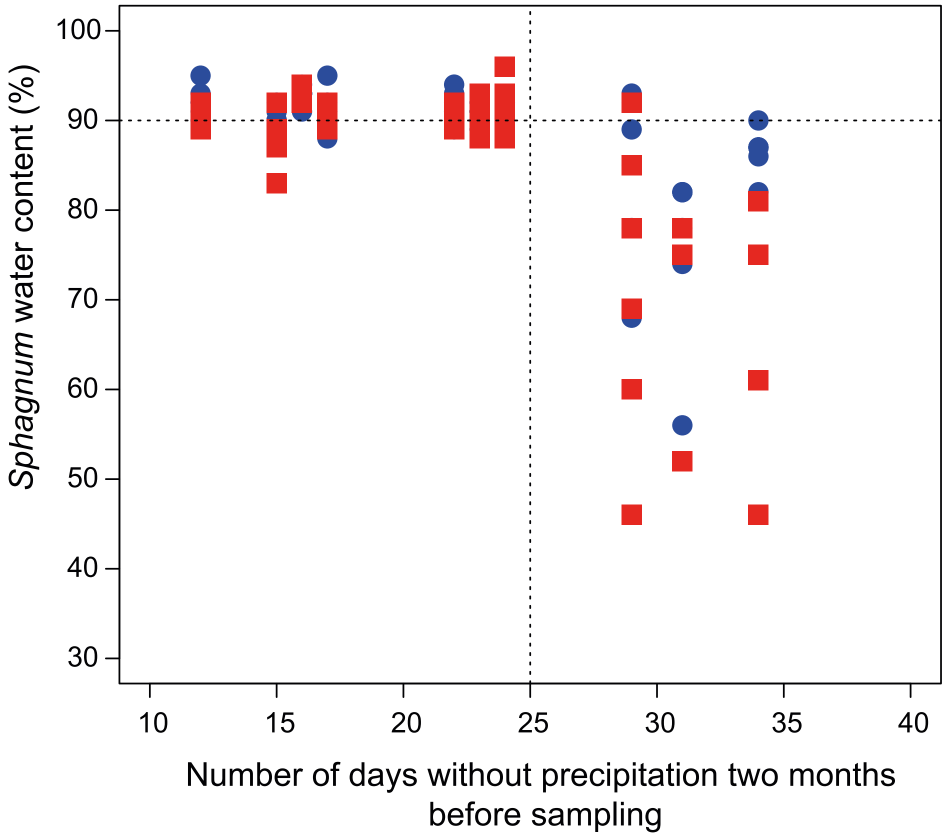


**Supplementary Figure S4| Relationships between *Sphagnum* water content and the number of days without precipitation during a two-months period before sampling.** *Sphagnum* water content (%) remains stable (90%) until 25 days without precipitation and then decreases down to 40-80% (data from June 2008 to June 2013, temporal trends are shown in Supplementary Fig. 2). Blue circles are control plots and red squares warmed plots.

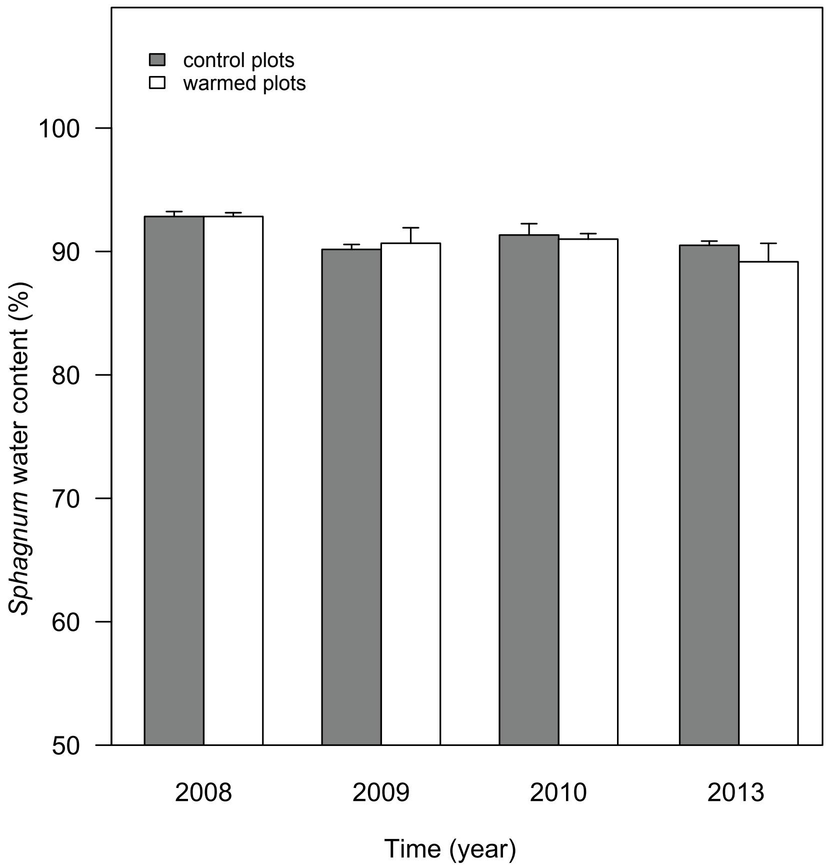


**Supplementary Figure S5| *Sphagnum* water content (%) in control and warmed plots on the dates of sampling each year.**

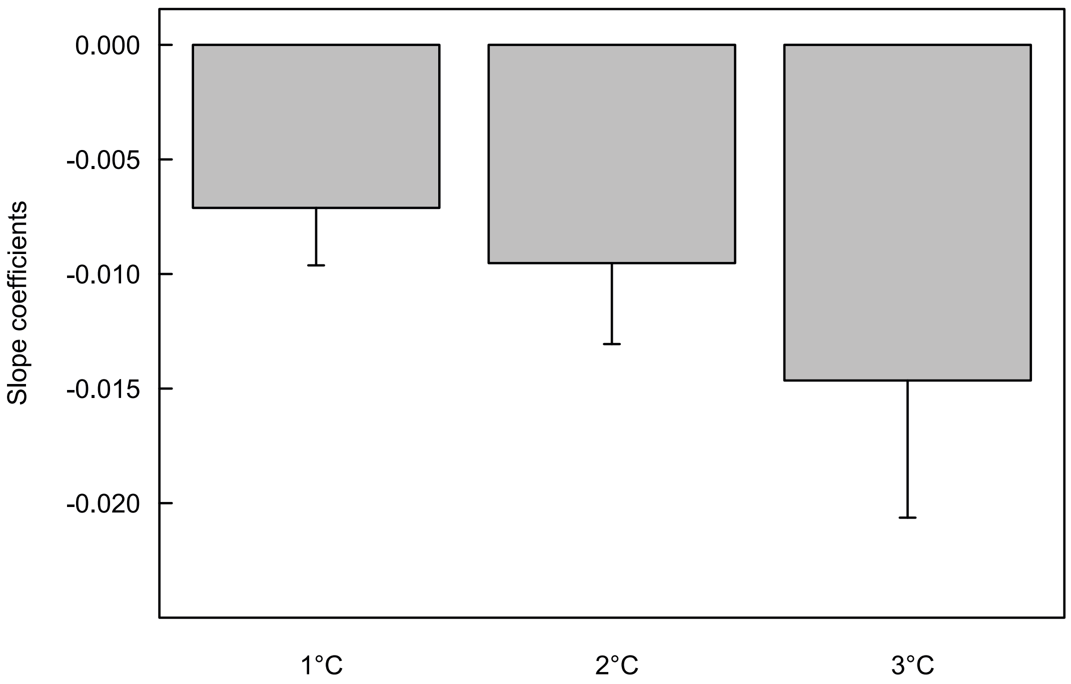


**Supplementary Figure S6| Slope coefficients from linear mixed effect models fitting the annual decrease of mixotrophic testate amoebae (Standardized effect size) as a function of the number of summer days with OTC warming effects higher than 1°C, 2°C and 3°C, respectively.** This shows that mixotrophic testate amoebae are more affected at higher temperature differences (3°C difference between OTC and control plots) compared to moderate warming of 1°C.

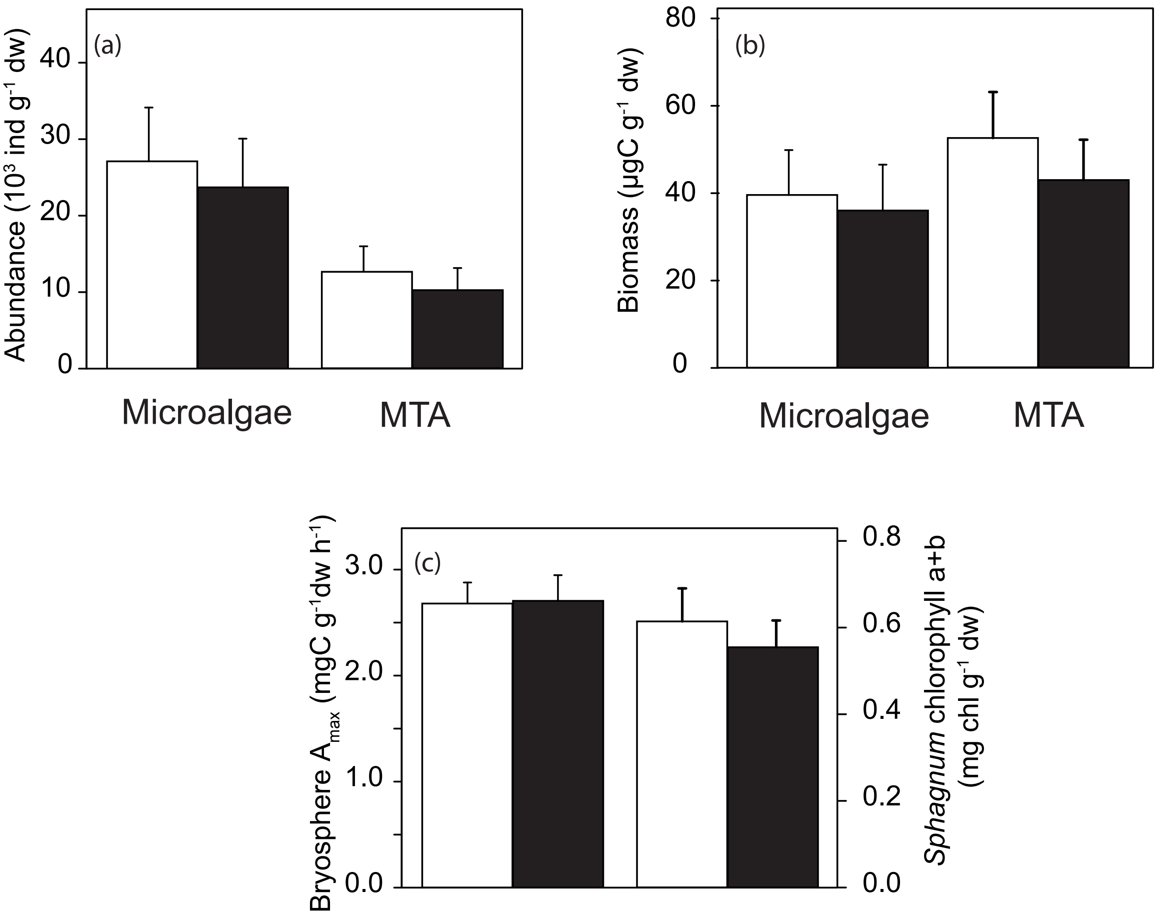


**Supplementary Figure S7| Initial conditions of the mesocosm experiment under controlled laboratory conditions.** (a) Abundance of microalgae (mean ± SEM) and mixotrophic testate amoeba (MTA; mean ± SEM) before the light manipulation experiment ('light' vs. 'dark' treatments). (b) Biomass of microalgae (mean ± SEM) and mixotrophic testate amoeba (MTA; mean ± SEM) before the light manipulation experiment ('light' vs. 'dark' treatments). (c) Bryosphere photosynthesis ($A_{max,\ bryo}$) and Bryosphere chlorophyll a+b content before the light experiment. White bars indicate light treatment and black bars full dark treatment. No significant differences were found between treatments.

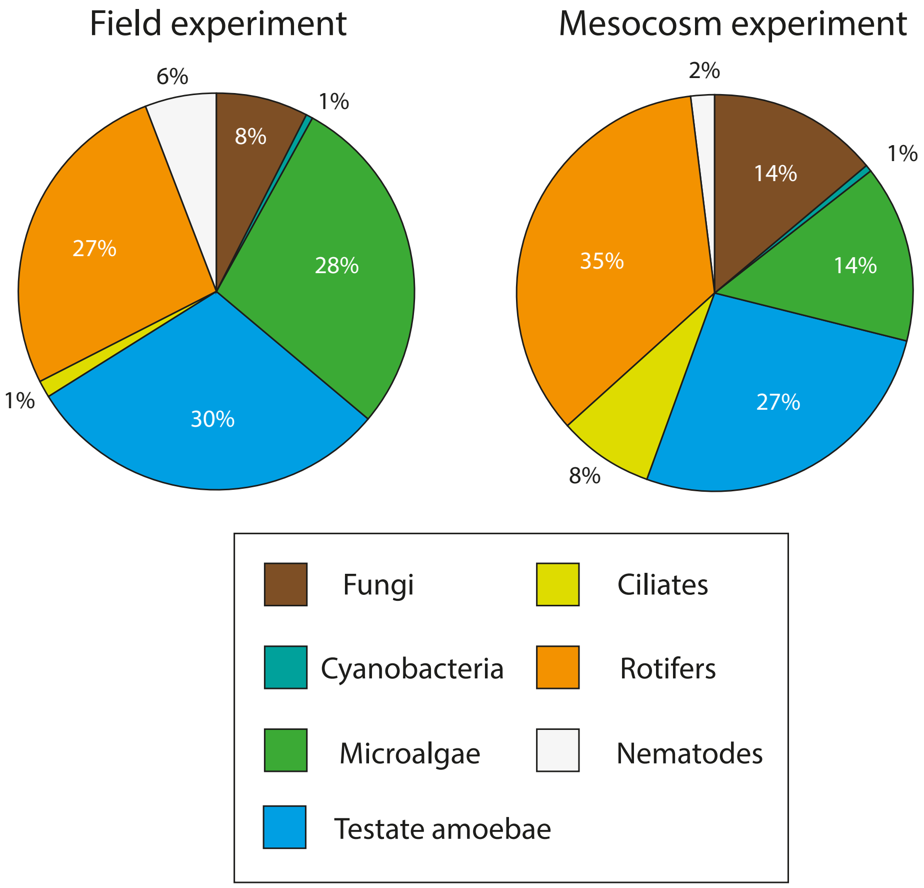


**Supplementary Figure S8| Structure of the microbial communities in the field and in the mesocosm experiment.** Relative biomass of fungi, cyanobacteria, microalgae, testate amoebae, ciliates, rotifers and nematodes in the field (control plots in 2008) and in the mesocosm experiment (before light treatment). Bacteria are not shown because they were not quantified in the mesocosms. Pie charts show a comparable microbial community structure between the field and mesocosm experiments. In addition, similar dominant species were found in both experiments. The Zygnematophyceae (i.e. desmids) *Cylindrocystis brebissonii* dominated microalgae communities, *Archerella flavum* and *Hyalosphenia papilio* dominated testate amoebae, while Bdelloids, *Colurella* sp., and *Lepedalla* sp. dominated rotifer communities, in both the field and the mesocosm experiment.

## Additional references


1. Pourriot, R. *Food and feeding habits of Rotifera*. (Arch. Hydrobiol. Beih, 1977).
2. Coûteaux, M.-M. Relationships between testate amoebae and fungi in humus microcosms. *Soil Biology and Biochemistry* **17,** 339–345 (1985).
3. Ogden, C. G. & Couteaux, M.-M. The Effect of Prédation on the Morphology of Tracheleuglypha dentata (Protozoa: Rhizopoda). *Archiv für Protistenkunde* **136,** 107–115 (1988).
4. Yeates, G. W. & Foissner, W. Testate amoebae as predators of nematodes. *Biol Fert Soils* **20,** 1–7 (1995).
5. Gilbert, D., Amblard, C., Bourdier, G. & André-Jean, F. Le régime alimentaire des thécamoebiens (Protista, Sarcodina). *L'année Biologique* **39,** 57–68 (2000).
6. Gilbert, D., Mitchell, E. A., Amblard, C., Bourdier, G. & Francez, A.-J. Population dynamics and food preferences of the testate amoeba Nebela tincta major-bohemica-collaris complex (Protozoa) in a Sphagnum peatland. *Acta protozoologica* **42,** 99–104 (2003).
7. Mieczan, T. Ciliates in Sphagnum peatlands: vertical micro-distribution, and relationships of species assemblages with environmental parameters. *Zoological Studies* (2009).
8. Jassey, V. E. J., Shimano, S., Dupuy, C., Toussaint, M.-L. & Gilbert, D. Characterizing the feeding habits of the testate amoebae Hyalosphenia papilio and Nebela tincta along a narrow 'fen-bog' gradient using digestive vacuole content and 13C and 15N isotopic analyses. *Protist* **163,** 451–464 (2012).
9. Jassey, V. E. J. *et al.* To What Extent Do Food Preferences Explain the Trophic Position of Heterotrophic and Mixotrophic Microbial Consumers in a Sphagnum Peatland? **66,** 571–580 (2013).
